# Notch4 activation aggravates NF-κB-mediated inflammation in HIV-1-associated nephropathy

**DOI:** 10.1242/dmm.040642

**Published:** 2019-12-17

**Authors:** Rajni Vaid Puri, Sireesha Yerrathota, Trisha Home, Jessica Y. Idowu, V. Praveen Chakravarthi, Christopher J. Ward, Pravin C. Singhal, Gregory B. Vanden Heuvel, Timothy A. Fields, Madhulika Sharma

**Affiliations:** 1 Department of Internal Medicine, University of Kansas Medical Center, Kansas City, KS 66160, USA; 2 The Jared Grantham Kidney Institute, University of Kansas Medical Center, Kansas City, KS 66160, USA; 3 Department of Pathology and Laboratory Medicine, University of Kansas Medical Center, Kansas City, KS 66160, USA; 4 Institute of Molecular Medicine, Feinstein Institute for Medical Research and Zucker School of Medicine at Hofstra-Northwell, New York, NY 11549, USA; 5 Department of Biomedical Sciences, Western Michigan University, Kalamazoo, MI 49008, USA

**Keywords:** HIVAN, Notch4, Inflammation

## Abstract

Notch pathway activation plays a central role in the pathogenesis of many glomerular diseases. We have previously shown that Notch4 expression was upregulated in various renal cells in human immunodeficiency virus (HIV)-associated nephropathy (HIVAN) patients and rodent models of HIVAN. In this study, we examined whether the Notch pathway can be distinctly activated by HIV-1 gene products and whether Notch4, in particular, can influence disease progression. Using luciferase reporter assays, we did not observe activation of the *NOTCH4* promoter with the HIV protein Nef in podocytes. Further, we observed upregulated expression of a gamma secretase complex protein, presenilin 1, but not Notch4, in podocytes infected with an HIV-1 expression construct. To assess the effects of Notch4 on HIVAN disease progression, we engineered Tg26 mice with global deletion of the Notch4 intracellular domain (*Notch4^dl^*), which is required for signaling function. These mice (*Notch4^d1^/Tg26^+^*) showed a significant improvement in renal function and a significant decrease in mortality compared to Tg26 mice. Histological examination of kidneys showed that *Notch4^d1^/Tg26^+^* mice had overall glomerular, tubulointerstitial injury and a marked decrease in interstitial inflammation. A significant decrease in the proliferating cells was observed in the tubulointerstitial compartments of *Notch4^d1^/Tg26^+^* mice. Consistent with the diminished inflammation, kidneys from *Notch4^d1^/Tg26^+^* mice also showed a significant decrease in expression of the inflammatory cytokine transcripts *Il-6* and *Ccl2*, as well as the master inflammatory transcription factor NF-κB (*Nfkb1* transcripts and p65 protein). These data identify Notch4 as an important mediator of tubulointerstitial injury and inflammation in HIVAN and a potential therapeutic target.

## Abstract (precis)

**Summary:** Notch4 activation contributes to the inflammation seen in HIV-associated nephropathy (HIVAN), and inhibition of Notch4 ameliorates inflammation and prolongs life in a mouse model of HIVAN.

## INTRODUCTION

Human immunodeficiency virus (HIV)-associated nephropathy (HIVAN) is a complication of HIV infection characterized by pathological changes in the kidney that include collapsing focal segmental glomerulosclerosis, tubular injury and dilatation, significant interstitial inflammation and tubuloreticular inclusions. HIVAN disproportionally affects individuals of African descent, largely due to high allele frequency of specific polymorphisms (Freedman and Langefeld, 2012; Papeta et al., 2011). With the advent of highly active anti-retroviral therapy (HAART), the incidence of HIVAN has greatly declined (Razzak Chaudhary et al., 2015). However, HIVAN remains a leading cause of end-stage renal disease (ESRD) in patients with HIV-1 infection, and HIVAN patients who reach ESRD experience excessive mortality compared with other ESRD patients (Gonzales et al., 2010; Li and Zhuang, 2014; Rachakonda and Kimmel, 2010; Razzak Chaudhary et al., 2015). Even in the HIV-infected patients who avoid HIVAN, chronic use of HAART itself is associated with nephrotoxicity, drug resistance and other serious complications. Thus, there is a need for alternative or combined therapies that may combat HIV-1-associated chronic kidney diseases and limit undesired effects.

Notch signaling is a developmental pathway that is required to orchestrate proper cell fate decisions, during embryogenesis and differentiation (Kopan and Ilagan, 2009; McCright et al., 2001), and has been implicated in HIVAN pathogenesis (Sharma et al., 2010, 2013). In mammals, there are four Notch receptors (Notch1, Notch2, Notch3, Notch4) and five ligands [jagged 1 (Jag1), jagged 2 (Jag2), delta-like 1 (Dll1), delta-like 2 (Dll2) and delta-like 4 (Dll4)]. Canonical Notch signaling is activated when a Notch ligand binds to its receptor, which initiates a series of proteolytic events. The final proteolytic event is mediated by presenilin-dependent gamma-secretase-like proteases, resulting in release of the Notch intracellular domain (NICD) and its translocation into the nucleus. In the nucleus, the NICD associates with a transcription factor, RBP-jk (also known as Rbpj and CBF-1), and activates the expression of members of the Notch effector protein family, Hairy enhancer of split (Hes and Hey) (Kopan and Ilagan, 2009).

Notch signaling is primarily known as a developmental pathway; however, post-developmental Notch activation has been associated with pathological conditions, including kidney disease (Arboleda-Velasquez et al., 2008; Bielesz et al., 2010; El Machhour et al., 2015; Sharma et al., 2011). Moreover, non-redundant roles of Notch signaling have been described (Cheng et al., 2007; Djudjaj et al., 2012). Notch1 signaling appears to play a major role in many glomerular diseases (Murea et al., 2010; Waters et al., 2008). We previously reported that Notch4 is highly upregulated in HIVAN in both glomerular and tubular cells, and that Notch inhibition by gamma secretase inhibitor (GSIXX, or dibenzazepine) can ameliorate the disease progression (Sharma et al., 2010, 2013). The specific role of Notch4 activation in HIVAN, and whether the increased expression is causative or a consequence of the disease, remains unanswered.

In the present study, we explored the role of Notch4 activation in HIVAN. We found that HIV-1 increased the gamma secretase activity but did not induce Notch4 activation in podocytes in culture. Further, Notch4 deletion in mice transgenic for HIV-1 proteins (Tg26 mice) resulted in significant protection from HIVAN, with a reduction in tubulointerstitial proliferation, inflammation and injury, preservation of renal function and a significant increase in survival.

## RESULTS

### Activation of the Notch signaling pathway by HIV-1

**Fig. 1.**
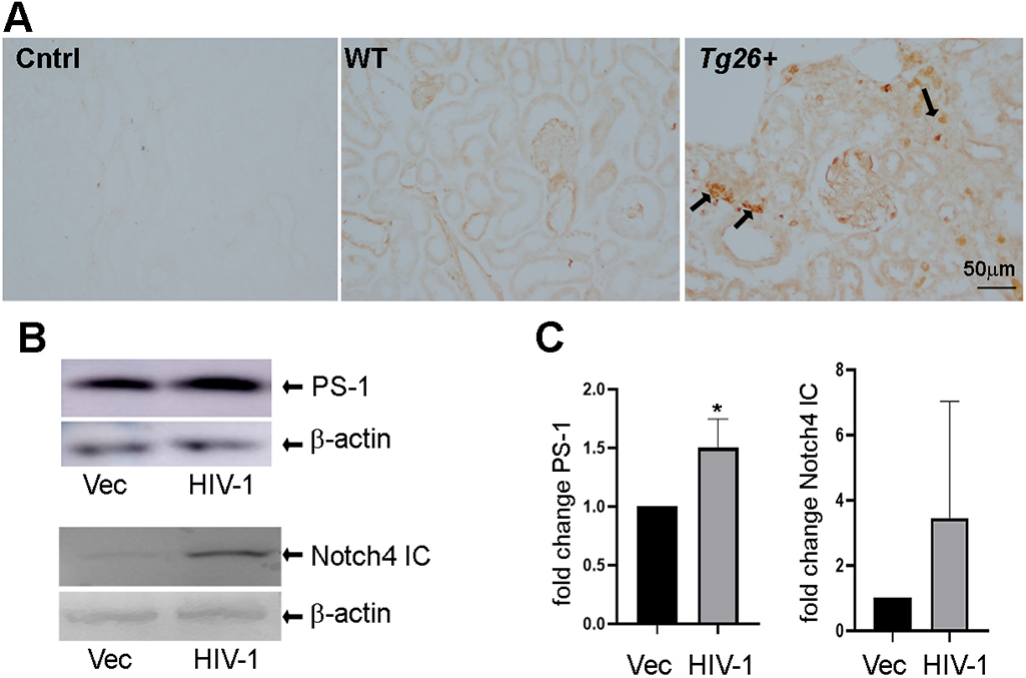
**HIV-1 induces activation of the Notch pathway.** (A) Immunohistochemistry (IHC) was performed for the presence of Notch4 on renal paraffin sections from 3-month-old wild-type (WT) or *Tg26^+^* mice. Arrows indicate interstitial cells positive for Notch4 expression in *Tg26^+^* mice compared to WT mice. The leftmost panel represents the no primary antibody control. (B) Differentiated immortal human podocytes were infected with empty vector (Vec) or HIV-1 construct followed by protein blots for the presence of presenilin1 (PS-1) and Notch4 intracellular (IC). (C) Protein blots were quantitated for PS-1 (*n*=3) and Notch4 IC (*n*=6) using β-actin (as a loading control), to which the values were normalized. Unpaired Student's *t*-test was used, and data are represented as fold change (*P<0.05).

The Tg26 mouse is a widely studied model of HIVAN in which all HIV-1 proteins except gag and pol are expressed (Dickie et al., 1991; Kopp et al., 1992). The heterozygous Tg26 mice (*Tg26^+^*) replicate HIVAN pathology, which is mainly due to the transgene expression in the kidneys (Bruggeman et al., 1997). We have previously shown that the Notch signaling pathway, specifically Notch4, is activated in several cell types in the kidney, including podocytes, parietal epithelial cells and renal tubular cells, in *Tg26^+^* mice and HIVAN patients (Sharma et al., 2010, 2013). In addition, we found that there are scattered Notch4-expressing cells in the interstitium (Fig. 1A, arrows). To determine whether Notch4 is a contributor to HIVAN pathogenesis in podocytes, we infected differentiated immortal human podocytes with either virions obtained from vector alone or an HIV-1 construct expressing seven of the nine HIV-1 genes (pNL4-3:ΔG/P-GFP). Western blots of lysates from these cells showed that the presence of HIV-1 genes significantly increased the expression of a gamma secretase complex component, presenilin 1 (PS-1; also known as Psen1), indicating that Notch signaling is activated. However, the activated signaling component of Notch4 [Notch4 intracellular (IC)] was not significantly increased compared to the vector-infected controls (Fig. 1B,C). These data indicate that HIV infection results in Notch pathway activation but may not involve activation of Notch4 in particular, in immortal differentiated podocytes *in vitro*.

### The HIV-1 gene product Nef does not induce *NOTCH4* promoter activity in podocytes *in vitro*

**Fig. 2.**
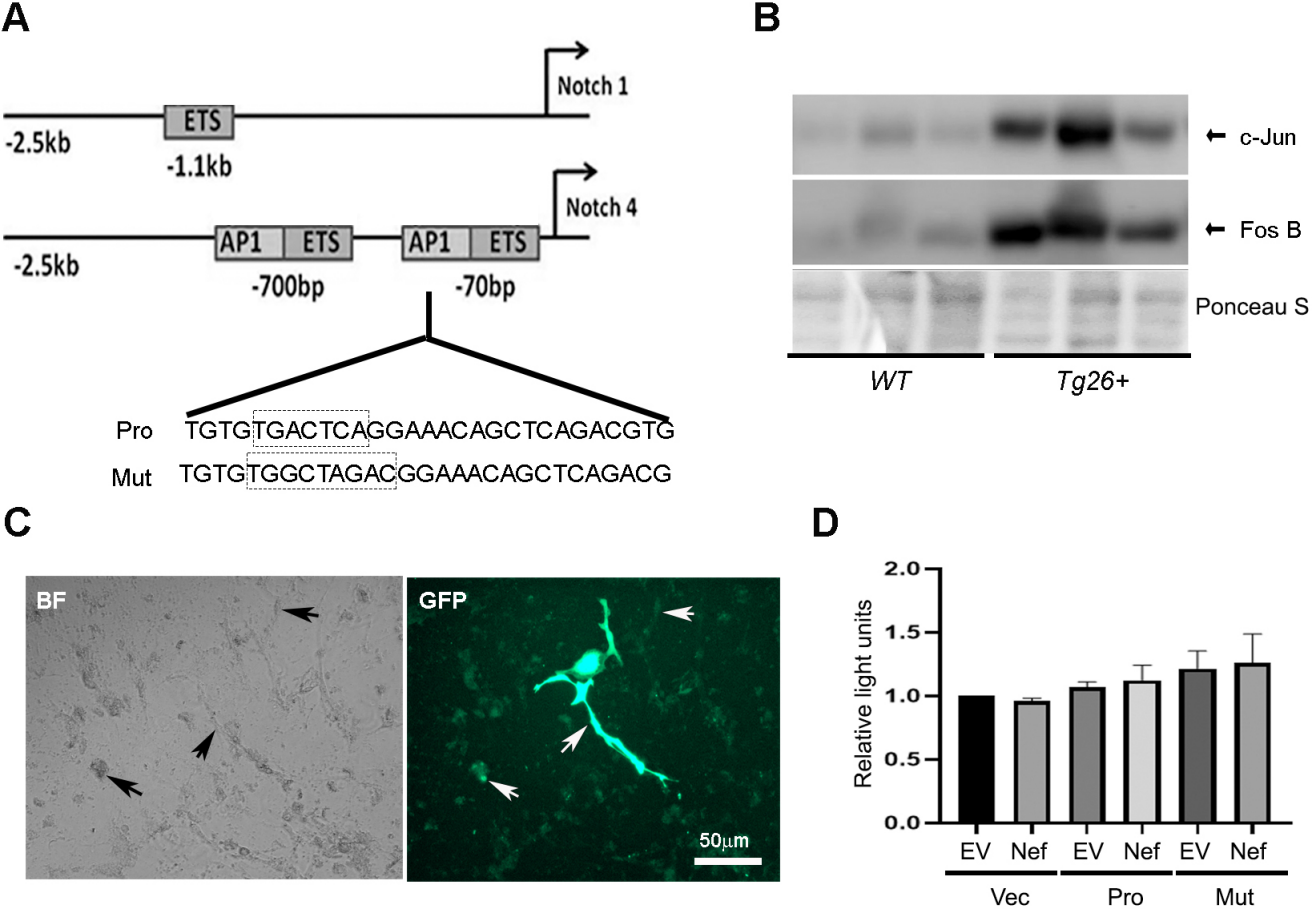
**HIV-1 gene products do not activate the Notch4 promoter in podocytes *in vitro*.** (A) Schematic showing promoter regions of *Notch1* and *Notch4*. The *Notch4* promoter contains two AP1 sites, one in close vicinity to the transcription start site [−70 base pairs (bp)]. The sequences involved in the intact AP1 binding site (Pro) and the mutant AP1 binding site (Mut) on the *Notch4* reporter construct are shown in the boxes below. (B) Renal tissue lysates from WT and *Tg26^+^* mice were used for protein blotting and probed for the presence of c-jun and fos B. Ponceau S was used to show loading. (C) Immortal differentiated podocytes were transfected using GFP-expressing vector. The transfection efficiency of podocytes is shown by cells expressing GFP. Arrows show cells with low and high GFP expression after 48 h (right panel). The left panel shows cells in brightfield (BF). (D) Dual luciferase reporter assays show *Notch4* transcriptional activity in podocytes transfected with empty vector (Vec), *Notch4* promoter reporter construct (Pro) or Notch4 promoter reporter construct with mutated AP1 site (Mut), with empty vector (EV) or *Nef* expression constructs (Nef). Reporter activity was normalized to renilla luciferase activity and expressed as relative light units (*n*=3). One-way AVOVA did not reveal any significance.

We next determined whether HIV-1 acts on Notch4 at the promoter level. The *NOTCH4* promoter at chromosome 6 has several *cis* elements, including a unique activator protein (AP1) motif, in proximity to the transcription start site. These unique sites are not present in the promoter regions of the *NOTCH1* receptor gene (Fig. 2A) (Wu et al., 2005). Interestingly, the HIV-1 proteins Nef, Tat, Vpr and Vpu activate and recruit AP1 in a variety of cells (Kumar et al., 1998; Varin et al., 2005, 2003). The AP1 transcription factor is a hetero- or homodimeric complex that contains members of the Jun (c-Jun, Jun-B and Jun-D) and Fos (c-Fos, Fos B) protein families. Stress-activated protein kinase/Jun N-terminal kinase (SAPK/JNK) signal transduction phosphorylates and activates Jun, which in turn activates AP1. To investigate the molecular mechanisms involved in Notch4 activation in HIVAN, we first verified that *Tg26^+^* mouse kidneys overexpressed both c-Jun and Fos B compared to the normal wild-type (WT) FVB mice (Fig. 2B). These observations imply that HIV proteins upregulate Notch4 transcription and may require AP1 activation to achieve this.

To determine whether there is any *NOTCH4* promoter activity in the immortal podocytes *in vitro*, reporter luciferase assays were performed. Podocytes were transfected with a GFP construct and ∼50% transfection efficiency was achieved, with most cells showing moderate expression of GFP and some very high expression at 48 h (Fig. 2C). Podocytes were then co-transfected with vector (Vec) alone or a luciferase construct containing the *NOTCH4* promoter that contained the AP1 binding site in close proximity to the transcription start site (Pro), or *NOTCH4* construct with a mutated AP1 site from TGACTCA to TGGCTAGAC (Mut) (Fig. 2A) (Wu et al., 2005), along with plasmids expressing *Nef* or vector alone. As shown in Fig. 2D, we did not achieve any significant *NOTCH4* promoter activity under any condition. This prompted us to verify our luciferase assays in human embryonic kidney (HEK) 293T cells with the same constructs. In HEK 293T cells, there was significant internal *NOTCH4* promoter activity and Nef further significantly enhanced this activity (Fig. S1). *NOTCH4* promoter activation was also seen in the presence of a separate gene, *Tat* (Fig. S1). In these cells, loss of AP1 binding did not diminish HIV-dependent activation of the *NOTCH4* promoter in the presence of either Nef or Tat (Fig. S1). These data indicate that the *NOTCH4* promoter is neither active nor activated by Nef in podocytes in culture.

### Notch4 deletion is not compensated by other Notch receptors

Although podocytes in culture did not appear to activate *NOTCH4* with HIV-1, this does not rule out the possibility of other glomerular and tubular cells exhibiting this phenomenon. Therefore, we opted for an *in vivo* approach, as opposed to a cell-based approach, to determine whether global deletion of Notch4 IC in the Tg26 mouse model would be functionally relevant. For this purpose, we bred HIV-1 transgenic mice (Tg26) with mice in which Notch4 IC was globally deleted (*Notch4^d1^*) (Krebs et al., 2000). Mice homozygous for the *Notch4^d1^* gene are viable and fertile and do not show any significant histological abnormalities (Krebs et al., 2000). These mice express extracellular Notch4 receptor but no Notch4 IC, which is required for the activation of Notch4 signaling (James et al., 2014). Breeding of *Notch4^d1^* and *Tg26^+^* mice resulted in mice with several genotypes. For our studies, we used four groups of mice: (1) normal *Notch4* alleles and no *Tg2*6^+^ transgene (WT control); (2) homozygous *Notch4^d1^* alleles with no *Tg26^+^* transgene (Notch4 control group; *Notch4^d1^*); (3) normal *Notch4* alleles and *Tg26^+^* transgene (Tg26 control group; *Tg26^+^*); and (4) *Notch4^d1^*mice with *Tg26^+^* transgene (*Notch4^d1^/Tg26^+^*)*.*

**Fig. 3.**
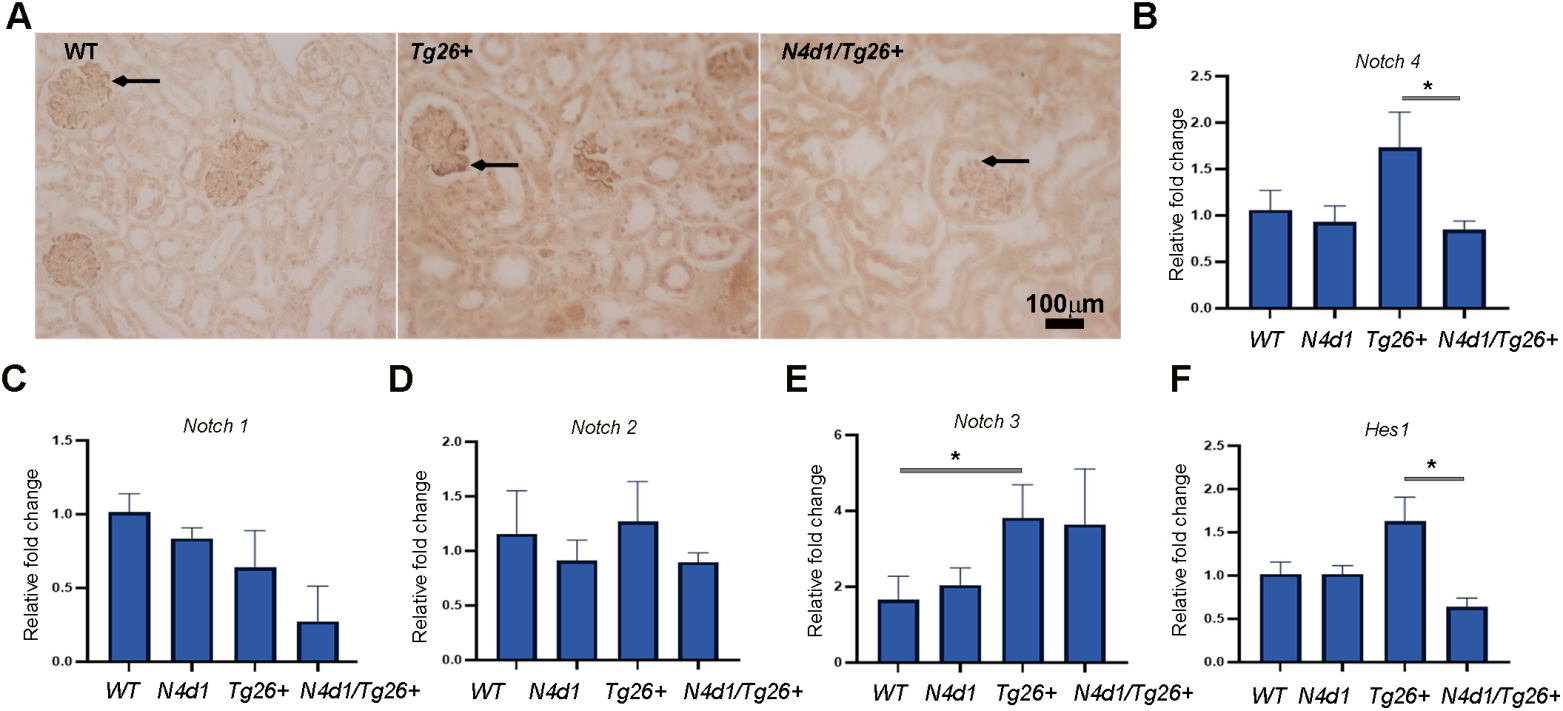
**Verification of Notch4 IC deletion and expression of other notch receptors in kidneys of *N4^d1^/Tg26^+^* mice.** (A) IHC was performed in paraffin kidney sections obtained from wild-type (WT), *Tg26^+^*, and *Tg26^+^*mice with Notch4 deletion (*N4d1/Tg26^+^*) for expression of Notch4. Arrows indicate glomerular expression. (B-F) Quantitative RT-PCR was performed for *Notch4*, *Notch1*, *Notch2*, *Notch3* and *Hes1* expression in RNA isolated from renal tissues from WT, *Tg26^+^*, *N4d1 and N4d1/Tg26^+^* mice. ΔΔCT method was used, and results were expressed as relative fold change (*n*=3 each). One-way ANOVA was used and the horizontal bars at the top of graphs show the significance (*P<0.05).

Notch4 IC deletion in the kidney was verified by immunohistochemistry (IHC) using an antibody that recognizes the truncated intracellular domain of mouse Notch4. As shown in Fig. 3A, when we compared the renal sections of *Tg26^+^*, WT and *Notch4^d1^/Tg26^+^* (*N4d1/Tg26^+^*), Notch4 expression was increased in *Tg26^+^* compared to the WT sections as expected. In *N4^d1^/Tg26^+^* sections, there was no specific staining observed (Fig. 3A, arrows). Quantitative reverse transcription PCR (RT-PCR) further verified reduction of *Notch4* expression in the *Notch4^d1^/Tg26^+^* mice (*P*<0.05) compared with the *Tg26^+^* mice (Fig. 3B)*.* Next, we determined whether expression of other Notch receptors might compensate for the loss of Notch4 IC. However, expression of the *Notch1*, *Notch2* and *Notch3* receptors was not affected by Notch4 IC deletion (Fig. 3C-E). A significant increase in *Notch3* expression in Tg26 mice was expected because Notch3 was another receptor that we have previously found to be elevated in Tg26 kidneys (Fig. 3E) (Sharma et al., 2010). Thus, the Notch receptor expression data suggest that any observed effects in mice are likely to be mediated specifically by deletion of Notch4. In *Notch4^d1^/Tg26^+^* mice, *Hes1* expression levels were reduced to normal levels, suggesting that Notch4 is primarily responsible for *Hes1* upregulation in *Tg26^+^* mice (*P*<0.05) (Fig. 3F).

### Notch4 deletion increases survival rate in *Tg26^+^* mice

**Fig. 4.**
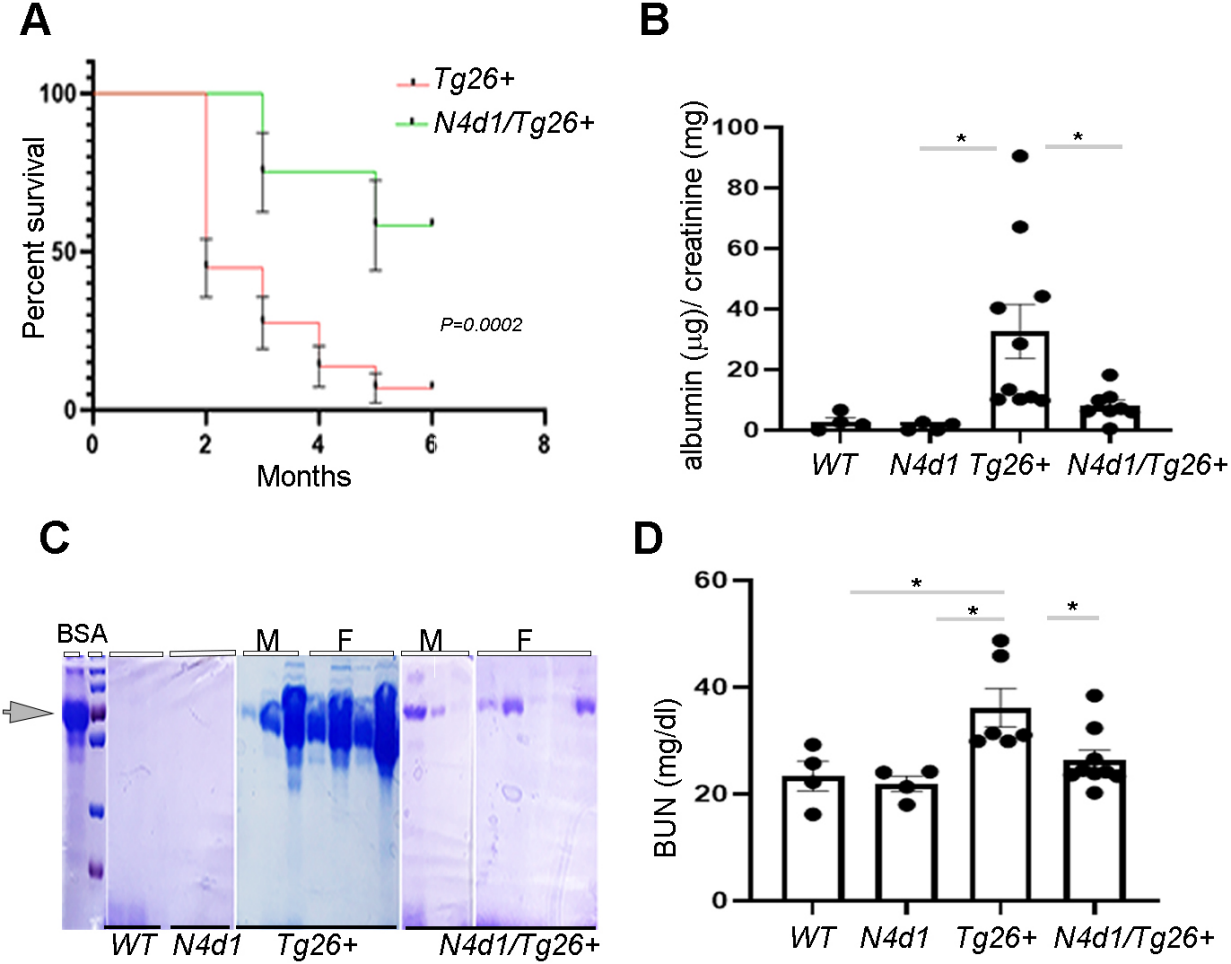
**Increased survival and protection of renal function in *N4^d1^/Tg26^+^* mice.** (A) *Tg26^+^* (*n*=37) and *N4^d1^/Tg26^+^* (*N4d1/Tg26+*) (*n*=14) mice were monitored for 6 months for any lethality issues; results are presented as percentage survival using Kaplan–Meyer curve (*P*=0.0002). (B) Urine albumin-to-creatinine ratio (µg/mg) was measured in all groups in 24 h collected urine samples (*P<0.05). (C) Proteinuria was also determined in 2 µl urine loaded onto 10% SDS-polyacrylamide gel for electrophoresis followed by staining with Coomassie Blue. The arrowhead indicates the 2 µl bovine serum albumin (BSA, 10 mg/ml) control band (M=males, F=females). (D) Serum blood urea nitrogen (BUN) levels were measured from all the groups and analyzed using one-way ANOVA followed by Tukey's multiple comparison test. The results are presented as mg/dl (*P<0.05).

To determine the effect of Notch4 IC deletion in *Tg26^+^* mice, all four groups of mice were monitored for 6 months after birth. As expected, WT and *Notch4^d1^* mice had 100% survival at 6 months (data not shown). *Tg26^+^* mice had low survival: ∼40% of the mice were alive at 2 months and ∼15% of the mice were alive at 6 months of age (Fig. 4A). Strikingly, Notch4 deletion significantly reduced mortality and increased survival in the *Tg26^+^* mice. Approximately 80% of the *Notch4^d1^/Tg26^+^* mice were alive at 3 months and ∼65% were alive at 6 months (Fig. 4A).

To determine whether increased survival is associated with improved renal function, albumin-to-creatinine ratio, proteinuria and blood urea nitrogen (BUN) levels were measured in all the groups at 3 months. Only 15-20% of the *Tg26^+^* mice survived up to 6 months; therefore, we restricted the analyses to 3-month-old mice. The albumin-to-creatinine ratio was significantly elevated in the 24 h collected urine of the *Tg26^+^* mice compared to WT and *Notch4^d1^* (*N4d1*) mice, and dropped to almost normal levels (*P*<0.05) in *Notch4^d1^/Tg26^+^* mice (Fig. 4B). Albumin levels were further assessed in 2 µl urine via Coomassie Blue staining. As shown (Fig. 4C), albumin was elevated in most *Tg26^+^* mice [both males (M) and females (F)] with some expected variability between males and females. In *Notch4^d1^/Tg26^+^* mice, albumin/proteinuria was markedly reduced*.* As expected, Tg26 mice had diminished renal function, as demonstrated by elevated serum BUN levels (Fig. 4D). Notably, the *Notch4^d1^/ Tg26^+^* mice had preserved renal function, with BUN levels comparable to those of WT and *Notch4^d1^* (*N4d1*) mice. These data indicated that Notch4 deletion slows the decline in renal function in *Tg26^+^* mice.

### Increased survival in Notch4-deleted *Tg26^+^* mice is associated with overall decrease in interstitial injury, glomerular injury and cell proliferation

**Fig. 5.**
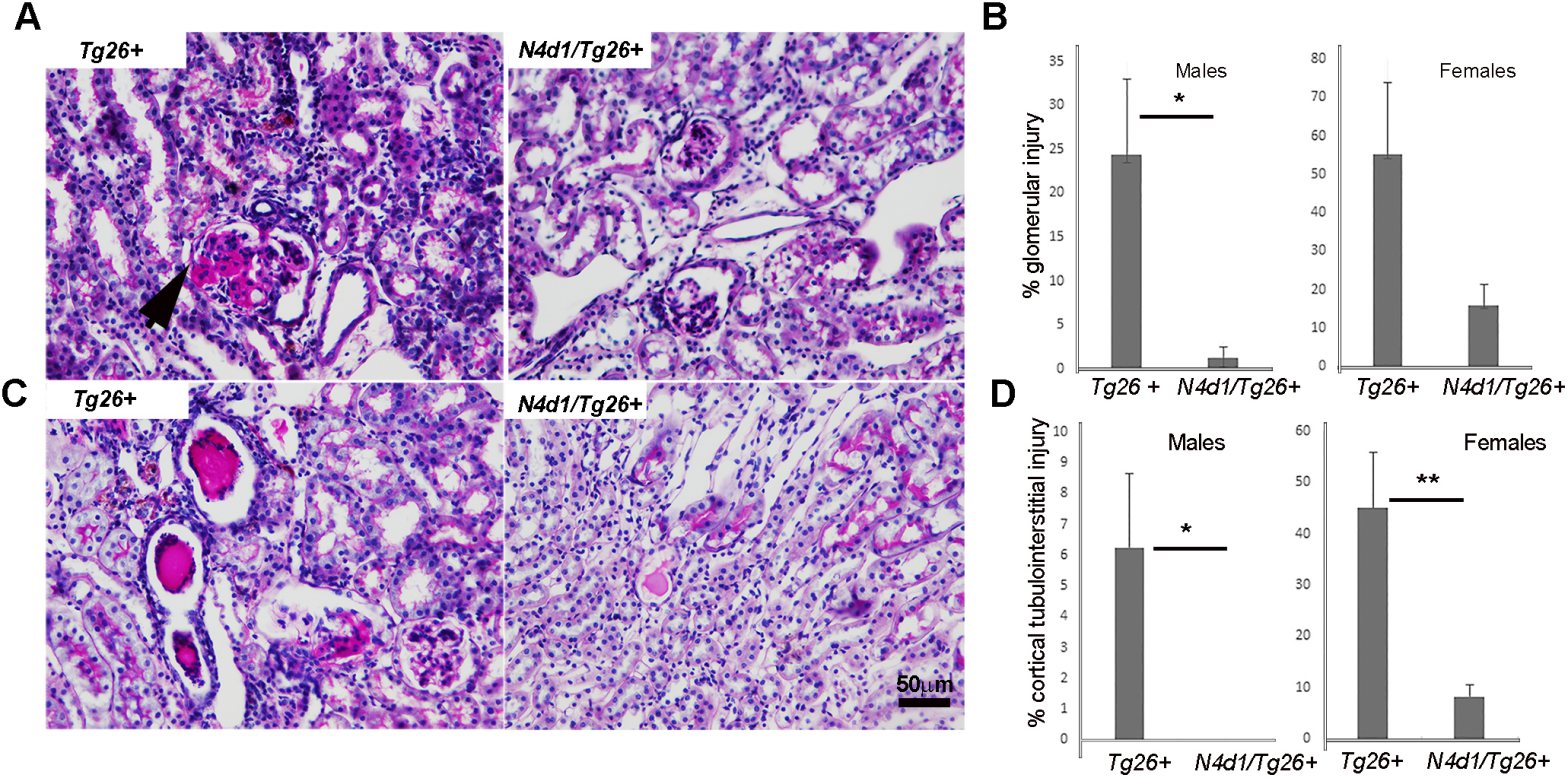
**Notch4 IC deletion rescues cortical tubulointerstitial and glomerular injury.** (A,C) Kidney paraffin sections from *Tg26^+^* and Notch4-deleted Tg26 (*N4d1/Tg26^+^*) mice (*n*=5 each) were subjected to periodic acid–Schiff staining and blindly evaluated for glomerular and tubulointerstitial injury. The arrowhead indicates sclerotic glomeruli. Note the hypercellularity in the *Tg26^+^* mice sections. (B,D) Percentage glomerular or tubulointerstitial injury was measured per kidney (*n*=5) for each group of mice. Statistical values were obtained using unpaired Student's *t*-test (*P<0.05, **P<0.01).

**Fig. 6.**
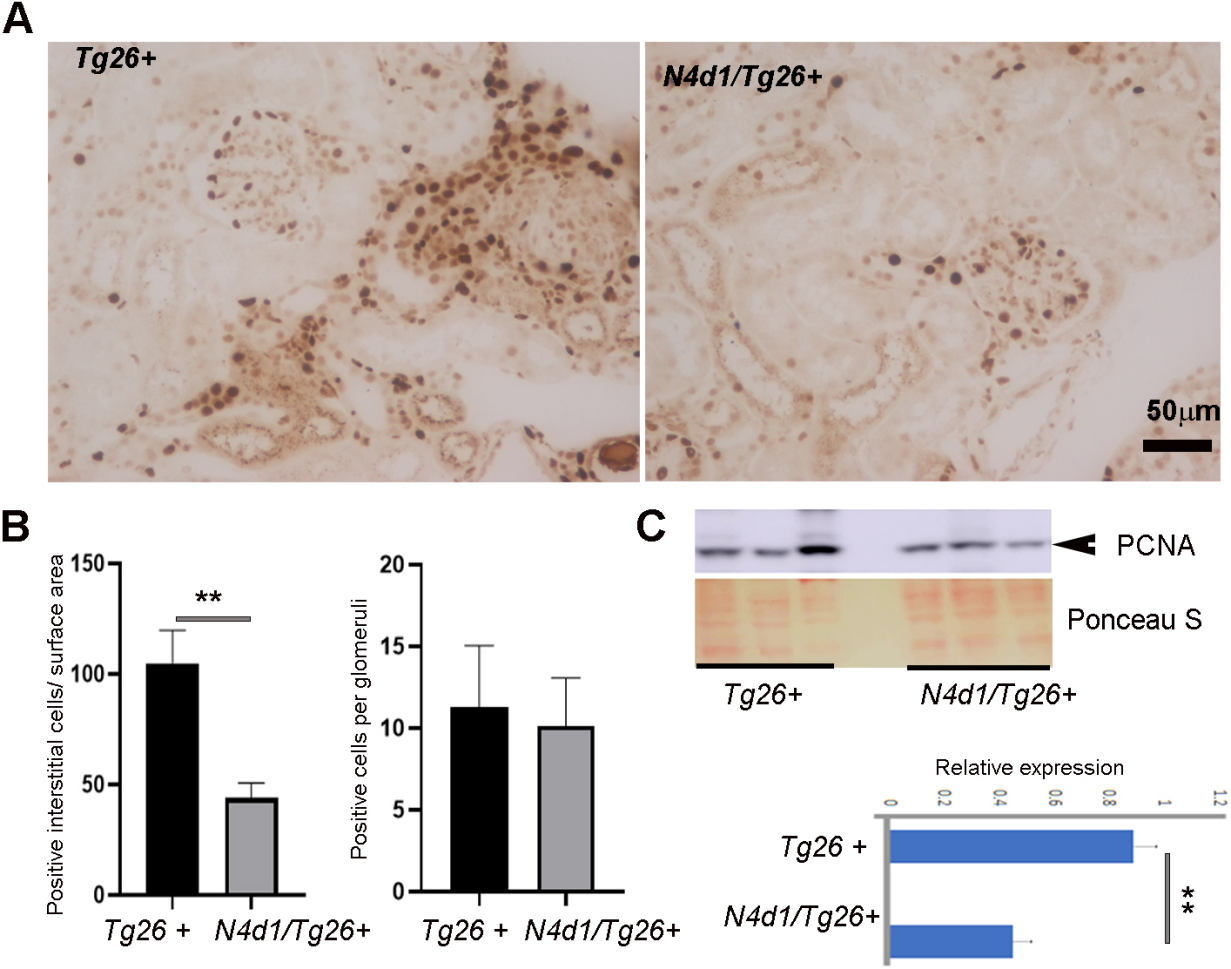
**Notch4 IC deletion is associated with decreased proliferating cells in the tubulointerstitial compartments.** (A) IHC was carried out for the presence of proliferative nuclear antigen (PCNA) in kidney sections from *Tg26^+^* and *N4d1/Tg26^+^* mice. (B) The total number of interstitial cells stained positive for PCNA was counted per field in about six random fields per group. Each group consisted of three male mice (*n*=3). The results are presented as number of PCNA-positive cells/surface area (left). The average number of PCNA-positive cells per glomerulus in about six random fields per group was also counted and averaged (*n*=3). The results are presented as average number of positive cells/glomerulus (right). (C) PCNA expression was determined via immunoblotting, using Ponceau S as a loading control, followed by semi-quantitative analysis of bands (lower panel) using ImageJ. Results are expressed as relative expression. Statistical values were obtained using unpaired Student's *t*-test (**P<0.01).

To further determine factors influencing the longevity of *Notch4^d1^/Tg26*^+^ mice, histological analysis was carried out. In both males and females, there was an improvement in overall renal pathology in *Notch4^d1^/Tg26*^+^ mice. A marked reduction in the percentage of glomerular injury [from 55.05±18.6 to 16.05±5.3 (females) and from 24.41±1.25 to 8.6±1.25 (*P*<0.05) (males)] was observed (Fig. 5B). Very mild tubulointerstitial injury was observed, if any, in the *Notch4^d1^/Tg26*^+^ male mice, and tubulointerstitial injury was significantly reduced in *Notch4^d1^/Tg26*^+^ females compared to *Tg26^+^* controls. The *Notch4^d1^/Tg26*^+^ mice (Fig. 5A,C, right panels) also had less interstitial inflammation compared to *Tg26^+^* controls (Fig. 5A,C, left panels). Overall proliferation was assessed by measuring proliferating cell nuclear antigen (PCNA), both in kidney lysates by western blotting and in sections by IHC (Fig. 6A-C). Intense PCNA-positive cells were seen in the tubulointerstitial compartments in *Tg26^+^* mice and were markedly reduced in *Notch4^d1^/Tg26*^+^ sections. The quantitation of PCNA-positive cells revealed that there was a reduction in PCNA-positive cells in the tubulointerstitial compartment but not in glomeruli (Fig. 6B). Western blotting for PCNA further showed an overall reduction in PCNA levels (*P*<0.01) (Fig. 6C).

### Notch4 deletion reduces inflammation in *Tg26^+^* mice via inactivation of inflammatory mediators, including NF-κB

**Fig. 7.**
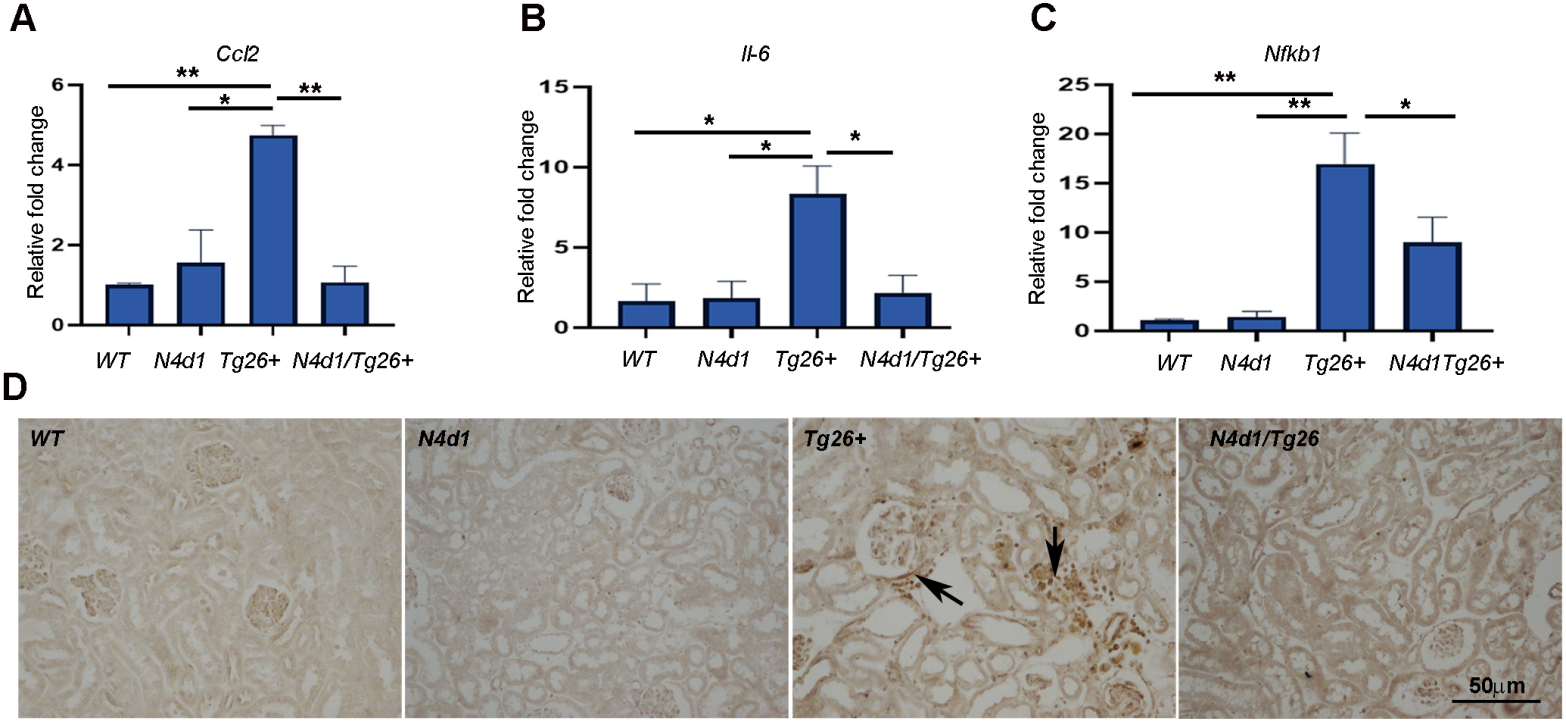
**Notch4 IC deletion results in a significant loss of inflammatory markers including NF-κB.** (A-C) RNA prepared from kidneys of WT, *N4d1*, *Tg26^+^* and *N4d1/Tg26^+^* mice (*n*=3 each) were subjected to quantitative RT-PCR analysis using primers for *Ccl2*, *Il-6* and *Nfkb1*. The results are expressed as relative fold change (*n*=3) (D) IHC for NF-κB (p65) was carried out; arrows show NF-κB-positive cells in the interstitial as well as in the glomerular compartments from *Tg26^+^* mice. Statistical significance was obtained using one-way ANOVA, followed by Tukey's multiple comparison test (*P<0.05, **P<0.01).

To characterize the effects of Notch4 deletion on inflammation, renal expression of the inflammatory cytokine monocyte chemoattractant protein-1 (MCP-1) transcript *Ccl2* and *Il-6* were measured by quantitative RT-PCR (Fig. 7A,B). MCP-1 is a chemoattractant involved in macrophage recruitment in renal injury and is known to be upregulated in human HIVAN (Kimmel et al., 2003). IL-6 is an inflammatory cytokine that may promote HIV gene expression in the kidney (O'Donnell et al., 1998). *Ccl2* mRNA levels were very low in the WT and *Notch4^d1^* mice. In *Tg26^+^*, renal expression of *Ccl2* was upregulated approximately 6-fold (Fig. 7A) (*P*<0.01). Notch4 deletion restored *Ccl2* expression to WT levels. A similar pattern was observed with *Il-6* (Fig. 7B).

NF-κB is a pro-inflammatory master transcription factor that is known to interact with HIV-1 long terminal repeat (LTR) and thus possibly contribute to HIVAN pathogenesis. NF-κB is a heterodimeric protein complex that consists of several different NF-κB family proteins (Bruggeman et al., 2001; Martinka and Bruggeman, 2006; Ross et al., 2005). We determined mRNA levels of p50 subunit (*Nfkb1*) and protein localization of NF-κB (p65) in the renal tissues of mice. Like the pattern described above for *Ccl2* and *Il-6*, there was significant increase in mRNA expression of *Nfkb1* in *Tg26^+^* mice, which dropped to normal levels in the *Notch4^d1^/Tg26^+^* mice (Fig. 7C) (*P*<0.05). Also, NF-κB protein levels were elevated in the glomerular and interstitial/infiltrating cells in *Tg26^+^* mice, and expression was markedly reduced in *Notch4^d1^/Tg26^+^* mice (Fig. 7D).

## DISCUSSION

Notch signaling has been relevant in several glomerular diseases, but, of the four Notch receptors, Notch4 has been the least investigated in this context. This is the first study to report a functional role for Notch4 activation specifically in HIVAN. Notch4 expression is generally restricted to the vasculature during development. However, in previous studies, we observed that Notch4 is upregulated in glomeruli and tubules in both *Tg26^+^* mice and human HIVAN (Sharma et al., 2010, 2013). For the current study, we used Tg26 mice with a *Notch4*-targeted deletion of exons 21 and 22, *Notch4^d1^* (Krebs et al., 2000). Notably, there was no change in expression of the other Notch receptors in the kidneys of these mice. Mice homozygous for this *Notch^d1^* allele are viable and fertile with slightly elevated blood pressure; the allele was later found not to be a true null, as a truncated transcript encoding most of the Notch4 extracellular domain was found to be expressed in these mice (James et al., 2014). However, the mice lack the intracellular domain of Notch4, which is required for signaling. Thus, the ameliorative effects of *Notch4^d1^* in the *Tg26^+^* mice reported here are most likely due to the absence of Notch4 signaling, which implies that Notch4 signaling facilitates HIVAN progression.

This study also investigated the nature of the link between HIV-1 and Notch4. In HEK 293T cells, Nef appeared to activate the *NOTCH4* promoter but activation was not dependent on the AP1 binding site. In cultured immortal podocytes, there was no *NOTCH4* promotor activity, and Nef did not appear to activate the *NOTCH4* promoter further and AP1 mutation did not alter this activity. The data suggested that Notch4 activation is not a major podocyte event in HIVAN. However, we cannot rule out the possibility of this event in other glomerular cells such as parietal epithelial cells or tubular cells, in which Notch4 was found to be activated in HIVAN.

The available literature suggests other modes of interaction of HIV-1 with the Notch pathway. One study employed a yeast two-hybrid system to demonstrate that Tat protein interacted with EGF-like repeats of Notch receptors (Shoham et al., 2003). However, *in vivo* evidence of these interactions is still lacking. Another study suggested an interaction of RBP-jk with the HIV-1 LTR promoter on two RBP-jk binding sites, showing RBP-jk to be a repressor of HIV-1 LTR, which was proposed to foster HIV latency (Tyagi and Karn, 2007). Since the Notch IC domain that is released upon Notch activation is known to convert RBP-jk from a repressor to a transcriptional activator, it is possible that Notch activation stimulates the HIV-LTR promoter and, hence, promotes entry into the lytic cycle. Thus, it is exciting to speculate that a positive feedback loop may exist whereby Notch and HIV-1 interact and mutually activate one another.

The Tg26 mouse model was generated by transgene expression of non-infectious HIV-1 DNA construct lacking the *gag* and *pol* genes. The homozygous mice are runted at birth and die within 40 days after birth. Heterozygous mice (*Tg26^+^*) are normal at birth but develop severe edema between 60 and 250 days of age and show characteristics of nephrotic syndrome. *Tg26^+^* mice develop ESRD within 100 days, with high levels of proteinuria. Kidneys show characteristic pathological changes, including focal segmental glomerulosclerosis, minor mesangial hyper cellularity, significant enlargement and vacuolization of glomerular parietal and visceral epithelial cells, microcystic tubular dilatations, protein casts, monocytic interstitial inflammation and interstitial fibrosis (Kopp et al., 1992). The improvements in mortality and renal function in the *Notch4^d1^/Tg26^+^* compared to *Tg26^+^* mice were accompanied by dramatic improvements in the pathological changes in the kidneys. There was significant improvement in glomerular and tubulointerstitial injury, and there was a dramatic decrease in overall inflammation. Relevant to this, kidneys from the *Notch4^d1^/Tg26^+^* mice showed a significant decrease in expression of the inflammatory cytokines *Il-6* and *Ccl2*, as well as the inflammatory transcription factor *Nfkb1*. These findings imply that Notch4 activation is one of the major determinants of increased inflammation in HIVAN. The overall improved proteinuria and glomerular lesions may be a secondary effect of improved tubulointerstitial inflammation and injury in these mice. Our study is supported by several other studies in which Notch silencing inhibited the NF-κB pathway in prostate cancer cells, pancreatic cancer cells and endothelial cells under inflammatory condition (Quillard et al., 2010; Wang et al., 2009; Zhang et al., 2017).

In our previously published expression data, Notch4 levels were found to be increased in podocytes, parietal epithelial cells and tubular cells (Sharma et al., 2013). Here, we find that the interstitial cells also express Notch4 in the nucleus, although the identity of these cells was not determined. Since many cells appeared to activate Notch4 upon HIV-1 infection, a global knockdown of Notch4 in the Tg26 mice was the approach taken in this study. However, to pinpoint the cell type responsible for the disease progression, conditional mouse models for cell specific deletion of Notch may be required. Also, single-cell sequencing may be illuminating. In the present study, we conclude that Notch4 deletion can significantly decrease inflammatory responses in HIVAN, in part by decreasing the tubulointerstitial injury mediated by NF-κB-dependent inflammation. Thus, blocking Notch4 activation in HIVAN may constitute a potential therapeutic approach.

## MATERIALS AND METHODS

### Animal care and protocol

*Tg26^+^* and *Notch4^d1^* mice (The Jackson Laboratory) used in this study were raised in accordance with the guidelines of the Guide for the Care and Use of Laboratory Animals of the National Institutes of Health. All the mice used were housed in micro-isolator cages, under aseptic and pathogen-free conditions, on a high-efficiency particulate air-filtered, ventilated rack. The protocols used in research were approved by the Institutional Animal Care and Use Committee of the University of Kansas Medical Center (Kansas City, KS, USA). *Tg26^+^* and *Notch4^d1^* were bred to obtain WT, *Notch4^d1^*, *Notch4^d1^/Tg26^+^* and *Tg26^+^* mice. As the *Tg26^+^* mice phenotype is restricted to FVB background, we first backcrossed *Notch4^d1^* (B6;129S1) mice onto FVB background for at least eight generations to obtain *Notch4^d1^* mice on FVB background. Mice were euthanized at 3 months for disease evaluation. Before euthanasia, 24 h urine was collected for evaluation of proteinuria and albumin-to-creatinine ratio. Blood was collected for serum isolation at the time of euthanasia. One half of a kidney from each mouse was fixed in 4% paraformaldehyde overnight, followed by a change in 70% ethanol. The second half and the second kidney were snap frozen for RNA isolation and protein lysates and stored at −80°C. A batch of mice was monitored for life expectancy for 6 months.

### Antibodies and reagents

The following antibodies were used: anti-presenilin 1 (1:1000, GenScript, A00881-40), anti-β-actin (1:1000, Sigma-Aldrich, A2228). anti-PCNA (1:100, Santa Cruz Biotechnology, sc-56) (c-Jun and Fos B, 1:500 each, Santa Cruz Biotechnology, 74543 and 8013), anti-NF-κB (p65) (1:100, Cell Signaling Technology, 8242) and anti-Notch4 (1:1000 for western blotting, 1:100 for IHC, Lifespan Biosciences, C9117).

### Renal function evaluation

Quantification of BUN in serum was performed using a Quantichrom Urea Assay Kit (Bioassay Systems), according to the manufacturer's protocol. Urinary albumin and creatinine were assayed using a mouse albumin ELISA kit (Bethyl Laboratories) and urinary creatinine assay kit (Cell Biolabs). Results were expressed as albumin-to-creatinine ratio. To get rid of any floating cells in urine, the urine was centrifuged and then 2 µl was loaded onto 10% SDS-polyacrylamide gel for electrophoresis followed by Coomassie Blue staining. Bovine serum albumin (BSA) (2 µl; 10 mg/ml) was used as a positive control.

### Histology

Kidneys were fixed in 4% paraformaldehyde overnight followed by 70% ethanol. The tissues were processed and embedded in paraffin at the core facilities of the University of Kansas Medical Center. Five-micrometer sections were stained by periodic acid–Schiff (PAS) and Hematoxylin and Eosin methods. Slides were examined in a blinded fashion by a renal pathologist (T.A.F.) and scored for tubulointerstitial disease and glomerular injury (*n*=5 each group). The percentage area with tubulointerstitial disease (enlarged, reactive tubular nuclei, tubular casts, tubular dilatation and interstitial fibrosis) was estimated by inspection for each section. For glomerular injury score, the number of glomeruli with disease (segmental and global sclerosis, adhesion to Bowman's capsule) was counted per section.

### Cell culture

All cell lines used were verified and were free of mycoplasma contamination. HEK 293T cell lines were maintained in Dulbecco's modified eagle medium/F-12 supplemented with 10% fetal bovine serum (FBS) and penicillin (100 U/ml)/streptomycin (130 µg/ml) (Pen/Strep) (Gibco by Life Technologies). Human immortal podocytes, a kind gift from Dr Moin Saleem (Saleem et al., 2002), were maintained at 33°C in a growth medium containing RPMI 1640 (Hyclone) [supplemented with 10% FBS, 1× Pen/Strep, 1× insulin-transferrin-selenium (GenDEPOT)] to promote expression of the SV40T antigen in the presence of 5% CO_2_ permissive conditions. To differentiate, 50% confluent cells were moved to 37°C (in 5% CO_2_) to inactivate SV40T antigen and allowed to differentiate for 7-10 days.

For Notch expression studies, replication-defective viral supernatants were prepared as described previously (Chandel et al., 2015; Husain et al., 2002). Briefly, green fluorescence protein (GFP) reporter gene (pEGFP-C1, from Clontech) was substituted in place of *gag/pol* genes in HIV-1 proviral construct pNL4-3, resulting in a pNL4-3:ΔG/P-GFP construct. This construct was used to produce high-titer virus stocks. The HIV-1 *gag/pol* and *VSV.G* envelope genes were used in trans using pCMV R8.91 and pMD.G plasmids, respectively, and were provided by Dr Didier Trono (Salk Institute, La Jolla, CA, USA) as kind gifts. As a negative control, pHR-CMV-IRES2-GFP-ΔB construct containing HIV-1 LTR and GFP empty expression vector was used. For production of infectious viral supernatants, HEK 293T cells were transiently transfected using an effectene kit (Qiagen), according to the manufacturer's instructions. After 48 h, when almost all (∼90%) cells appeared positive for GFP (∼72 h), the medium was collected and filtered with 0.45-μm syringe filters. For infections, 1.5 μl polybrene (EMD, Millipore) was added per 3 ml of virus medium (8-10 µg/ml) and added dropwise to the differentiated podocytes growing on 10 cm^2^ plates. Normal culture medium (7 ml) was added after 4 h and cells were used after 72 h, when 60-70% of the podocytes appeared GFP positive.

### Luciferase assays

Podocytes were seeded into 24-well plates and allowed to reach 50% confluency at 33°C, after which they were moved to 37°C to differentiate for 7 days. Co-transfections using pcDNA3.1SF2-*Nef* or empty vector [kind gifts from Dr Ashok Chauhan (Mehla et al., 2010; Zhang et al., 2010)] were performed, along with *NOTCH4* promoter reporter construct with or without intact AP1 binding sites. The *NOTCH4* promoter reporter construct was kindly provided by Dr Emery Bresnick (University of Wisconsin–Madison, Madison, WI, USA). Transfections were performed using Lipofectamine RNAiMAX Reagent (Invitrogen), according to the recommended procedure. Renilla vector construct pGl4.70 (*hRluc*) was used in each transfection to control for internal luminescence (Promega). After 48 hours of incubation, firefly and renilla luciferase activities were detected with a dual-luciferase reporter assay (Promega), according to the manufacturer's protocol.

### Quantitative RT-PCR

Total RNA was isolated from tissues using Trizol (Life Technologies), using the manufacturer's protocol. RNA concentration and purity were determined by nanodrop analysis. Complementary DNA (cDNA) was prepared from 2 μg total RNA using a High-Capacity Reverse Transcription Kit (Thermo Fisher Scientific). Quantitative RT-PCR was performed using Power SYBR Green Master Mix (Applied Biosystems). Results were normalized to 18S ribosomal RNA (rRNA) expression and calculated by the comparative ΔΔCT. Briefly, Ct values for the housekeeping gene (*Rn18s*), as well as for the gene of interest, were obtained for all samples (WT control, *Notch4^d1^*, *Tg26^+^* and *Notch4^d1^/Tg26^+^*)*.* Delta Ct (ΔCt) values for the samples were then calculated by subtracting the respective housekeeping gene value from the Ct value of the samples. Delta Ct values of the WT controls (*n*=3) were then averaged to obtain an average ΔCt value. The average ΔCt value of the WT controls was then subtracted from the ΔCt values of all the samples including WT controls to obtain the ΔΔCt value. Relative fold gene expression was then calculated using the 2^−ΔΔCT^ formula for each control or test sample. Values from each group were averaged and presented as relative fold gene expression as previously shown (Chakravarthi et al., 2018). The primers used for quantitative analysis of the various genes are listed in Table 1.

**Table 1.**
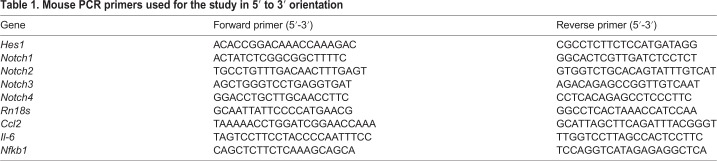
**Mouse PCR primers used for the study in 5′ to 3′ orientation**

### Cell lysates and western blots

Podocytes after infections were lysed for western blotting. Cells were washed three times with ice-cold PBS and lysed with RIPA buffer [137 mM NaCl, 50 mM Tris HCl pH 7.5, 12 mM EDTA, 1% IGEPAL and complete protease inhibitor (Thermo Fisher Scientific)]. Kidney tissues (fresh or frozen) were severed into small pieces and lysed in RIPA buffer with protease inhibitors using 50-80 strokes with a dounce homogenizer as shown previously (Idowu et al., 2018). Quantification of protein for cell or tissue lysates was performed with BCA protein assay (Bio-Rad, Hercules, CA, USA). Freshly prepared whole-cell extracts (50-100 μg) were solubilized in 4× NuPage (Novex) sample buffer [containing 25% tris(2-carboxyethyl) phosphine (TCEP)]. Samples were heated to 65°C for 10 min and then electrophoresed on 10% SDS-polyacrylamide gels, which were subsequently transferred to polyvinylidene difluoride (PVDF) membranes. Blocking of immunoblots was performed in 5% non-fat dry milk, in PBST (PBS containing 0.1% Tween 20), for 1 h, at room temperature (Betha et al., 2007). Blots were then washed with PBS, followed by overnight incubation with appropriate dilutions of primary antibodies. Blots were washed three times with PBST, at room temperature, and incubated with secondary antibodies (Vector Laboratories) (1:5000 dilution in blocking solution), at room temperature, for 1 h. The bound secondary antibody was detected by chemiluminescence (Western Lightning Plus ECL, Perkin Elmer).

### IHC

IHC was performed as described previously (Sharma et al., 2013). Kidney sections from WT, *Tg26^+^*, *Notch4^d1^* and *Notch4^d1^/Tg26^+^* mice were deparaffinized with xylene and hydrated with different grades of ethanol. Further, these sections were treated and boiled in citrate buffer (10 mM sodium citrate, 0.05% Tween 20, pH 6.0) and cooled to room temperature. Sections were incubated for 30 min with 3% hydrogen peroxide to block endogenous peroxidase activity, washed in PBS and blocked with 10% normal serum (from the species the secondary antibody was raised in, in PBS) for 1 h, followed by incubation for 1 h with primary antibodies in a humidified chamber. After washing slides three times with PBS, they were incubated for 1 h in 1:400 diluted biotin-conjugated secondary antibodies (Vector Laboratories) for IHC and fluorescein/Texas Red-conjugated antibodies for immunofluorescence (IF). Slides were washed four times in PBS for 5 min each. For IF, the slides were mounted using Vectashield (Vector Labs). For IHC analysis, the slides were further incubated with avidin-biotin-peroxidase complex (ABC Elite, Vector Laboratories), which was detected with diaminobenzidine (DAB, Sigma-Aldrich). Tissue sections were then dehydrated with graded ethanols and mounted with permount (Fisher Scientific, Pittsburg, PA, USA). Slides were viewed on a Nikon Eclipse 80i upright microscope.

### Statistics

Data are expressed as mean±s.e. Statistical significance was measured by unpaired Student's *t*-test for comparison between control and test groups. One-way ANOVA was performed to compare more than two groups, followed by Tukey's multiple comparison test. *P*<0.05 was considered significant.

## Supplementary Material

Supplementary information
