# Supplementary material for: Notch4 activation aggravates NF-κB-mediated inflammation in HIV-1-associated nephropathy
Source: Dis Model Mech. 2019 Dec 17;12(12):dmm040642. doi: 10.1242/dmm.040642 (PMC6918754; doi:10.1242/dmm.040642)
Supplement: Supplementary information [file dmm-12-040642-s1.pdf]

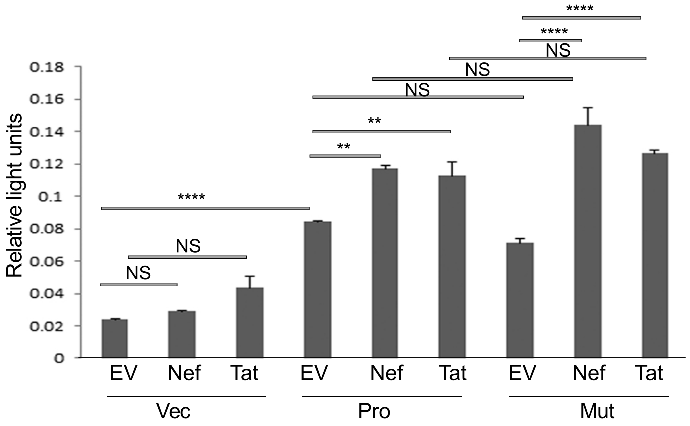

Figure S1

HEK 293T cells were seeded into 6 well plates and allowed to reach 70% confluency at 37^{0}C. Co-transfections using Nef/Tat or empty vector (EV) constructs were performed, along with vector alone (Vec), Notch4 promoter reporter construct with intact AP1 binding sites (Pro) or with a Notch4 promoter reporter construct without intact AP1 binding sites (Mut). Transfections were done using effectene kit (Qiagen Inc.) according to the manufacturer's instructions. Renilla was used in each transfection to control for internal luminescence. After 24 hrs. of incubation, the firefly and renilla luciferase activities were detected with a dual-luciferase reporter assay (Promega) as relative light units. On-way ANOVA was followed by post hoc Tukey’s multiple comparisons (*P<0.05) (**P<0.005) (****P<0.0001).

Disease Models & Mechanisms • Supplementary information
